# Veterinary Medical Association of New Jersey

**Published:** 1890-05

**Authors:** W. H. Cooper

**Affiliations:** Secretary


*Secretary.*

---

*Veterinary Medical Association of New Jersey.*—The sixth annual meeting of the *Veterinary Medical Association of New Jersey* met at Trenton, N. J., on Thursday, April 10th, with twenty-five members present. The newly-elected officers are: President, J. W. Hawk, of Newark; first vice-president, H. Bradshaw, of Trenton; second vice-president, W. W. Curry, of Jersey City; secretary, W. H. Cooper, of Trenton; treasurer, B. F. King, of Little

Silver; board of trustees, W. B. E. Miller, Camden; J. C. Dustan, Morristown; R. R. Letts, Hoboken; L. P. Hurley, Hopewell; W. Runge, Newark. Reports from Committee of Agriculture, Registration and Legislation took up considerable time. Dr. Miller read a paper on "Tuberculosis," by Fleming. R. T. Churchill, of Seacaucus, graduate of New York; Theo. DeClyne, of New Durham, graduate of Columbia, were elected active members, and three proposed for next meeting. Seven delegates or visitors from other States were present. The rest of the day was taken up in business. Our delegates for Pennsylvania for the year are J. C. Higgins and R. E. Stanwood; for New York, R. R. Letts, J. Huilson; Maryland, W. B. E. Miller, A. Brown.

W. H. Cooper,
*Secretary.*

